# Application of Referencing Techniques in EEG-Based Recordings of Contact Heat Evoked Potentials (CHEPS)

**DOI:** 10.3389/fnhum.2020.559969

**Published:** 2020-12-02

**Authors:** Malte Anders, Björn Anders, Matthias Kreuzer, Sebastian Zinn, Carmen Walter

**Affiliations:** ^1^Institute of Clinical Pharmacology, Goethe University, Frankfurt am Main, Germany; ^2^Department for Human Experimental Pain Models, Fraunhofer Institute for Molecular Biology and Applied Ecology (IME), Branch for Translational Medicine and Pharmacology (TMP), Frankfurt am Main, Germany; ^3^Department of Anesthesiology and Intensive Care, School of Medicine, Technical University of Munich, Munich, Germany; ^4^Department of Anesthesiology, Intensive Care Medicine and Pain Therapy, School of Medicine, University Hospital Frankfurt, Goethe University, Frankfurt am Main, Germany

**Keywords:** electroencephalography (EEG), EEG reference choices, event-related potentials (ERP), independent component analysis (ICA), pain research, contact heat evoked potentials (CHEPS)

## Abstract

Evoked potentials in the amplitude-time spectrum of the electroencephalogram are commonly used to assess the extent of brain responses to stimulation with noxious contact heat. The magnitude of the *N*- and *P*-waves are used as a semi-objective measure of the response to the painful stimulus: the higher the magnitude, the more painful the stimulus has been perceived. The strength of the *N*-*P*-wave response is also largely dependent on the chosen reference electrode site. The goal of this study was to examine which reference technique excels both in practical and theoretical terms when analyzing noxious contact heat evoked potentials (CHEPS) in the amplitude-time spectrum. We recruited 21 subjects (10 male, 11 female, mean age of 55.79 years). We applied seven noxious contact heat stimuli using two temperatures, 51°C, and 54°C, to each subject. During EEG analysis, we aimed to identify the referencing technique which produces the highest *N*-wave and *P*-wave amplitudes with as little artifactual influence as possible. For this purpose, we applied the following six referencing techniques: mathematically linked A1/A2 (earlobes), average reference, REST, AFz, Pz, and mathematically linked PO7/PO8. We evaluated how these techniques impact the *N*-*P* amplitudes of CHEPS based on our data from healthy subjects. Considering all factors, we found that mathematically linked earlobes to be the ideal referencing site to use when displaying and evaluating CHEPS in the amplitude-time spectrum.

## Introduction

To assess an individual’s sensitivity to pain, standardized painful stimuli that activate the A and C fibers in the human body are applied to the surface of the skin. A variety of techniques, including thermal, laser, mechanical (e.g., flat tip probes), or electrical stimulation are commonly used to evoke pain responses in the subject’s brain (Iannetti et al., 2013; Oh et al., 2015; Wulf et al., 2017; Albu and Meagher, 2019; Lefaucheur, 2019). The extent of the response strongly correlates with how “painful” the individual subjectively rates the applied stimulus; a good correlation between a visual analog scale (VAS) pain score communicated by the subject and the amplitude of the noxious contact heat evoked potentials (CHEPS) can usually be detected (Roberts et al., 2008). Parameters of interest are, for example, *N*- and *P*-waves [the lowest negative (N) or highest positive (P) peak in the average EEG amplitude-time spectrum after the stimulus] or the *N-*wave or *P*-wave delay (the latency from the stimulus to the respective peak). From a physiological point of view, the brain response to the noxious contact heat stimulus that is detectable in the electroencephalogram (EEG) is not merely described by a single feature of the subject’s brain activity but is somewhat derived from a complex combination of components that, if put together, result in the recorded amplitude (Ploner and May, 2018). Thus, the amplitude or the power of the response is also highly dependent on a variety of other factors which include, but is not limited to: (i) the individual’s subjective perception of how “painful” the stimulus is; (ii) the vigilance of the subject; (iii) the stimulation technique; (iv) the time interval between the stimuli and habituation occurring; (v) the exact placement of the recording electrodes; (vi) preprocessing steps (e.g., bandpass filtering) and any artifact rejection in the EEG analysis; and (vii) the reference site. Furthermore, it is feasible that the CHEPS response to the same stimulus by the same subject may vary when measured by different laboratories or by different scientists. After performing the experiments, the EEG data is usually processed for further analysis, hence, decisions regarding preprocessing, artifact rejection, and the choice of reference site need to be made. This study will demonstrate that the reference electrode site influences the CHEPS amplitude in the amplitude-time spectrum of the EEG. Thus, the referencing technique to be used for an EEG-based analysis of CHEPS should be carefully chosen during the study design phase. CHEPS between subject groups or different studies cannot be compared when different referencing techniques have been used. As referencing is a linear step, it can, fortunately, be changed after the EEG recording, irrespective of which reference site was initially defined (Dong et al., 2017). Theoretically, the reference site should be chosen as an “electrically neutral point” somewhere on the subject’s body, however, this is practically impossible (Kayser and Tenke, 2010). This study evaluated which reference site—considering the theoretical requirements—provides the best results in the recording and analysis of CHEPS.

## Materials and Equipment

### Parameters of Interest and Selection of Reference Sites

We extracted the following parameters from our EEG-data that can be analyzed after stimulation with noxious contact heat:

(i) *N-*wave: the lowest negative peak in the average EEG waveform in the amplitude-time spectrum across all seven trials for each stimulation temperature found between 250 ms to 550 ms after stimulation onset.
(ii) *P*-wave: the highest positive peak in the average EEG waveform in the amplitude-time spectrum across all seven trials for each stimulation temperature found between 550 ms to 800 ms after stimulation onset.
(iii) *N*-*P*-wave: the difference in amplitude between the *N-*wave and the *P*-wave.
(iv) *N-*wave delay: the latency between the onset of stimulation and *N-*wave.
(v) *P*-wave delay: the latency between the onset of stimulation and *P*-wave.
(vi) *N*-*P*-wave duration: the duration/latency between the *N-*wave peak and the *P*-wave peak.

We identified the following reference sites which we then further evaluated in this study for suitability in measuring CHEPS:

(i) Mathematically linked earlobes A1/A2, where the mathematical average of the earlobe electrodes (A1 and A2), according to the 10-10-system, was calculated and then subtracted from each individual EEG recording electrode (Jurcak et al., 2007).
(ii) AFz, where the single frontal electrode at position AFz, according to the 10-10-system, was used as a reference site (Jurcak et al., 2007).
(iii) Pz, where the central parietal electrode Pz, according to the 10-10-system, was used as a reference site (Jurcak et al., 2007).
(iv) Average reference, where the mathematical average of all EEG recording electrodes was calculated and then subtracted from each individual EEG recording electrode (Nunez, 2010).
(v) Reference Electrode Standardization Technique (REST), or Infinity Reference, where a virtual reference location at infinity was calculated (Yao, 2001). This reference-free approach assumed that the source of the EEG signal at each electrode location was the same, regardless of which reference was used. For REST, a lead field matrix needed to be calculated, that, in a linear relationship, routed the specific source to its measuring (electrode) location on the head. In practice, REST relied heavily on the head model that was used to calculate the reference signal; this could lead to biases and inaccuracies if the head model did not perfectly match the real-world scenario (Nunez, 2010).
(vi) Mathematically linked PO7/PO8, where the mathematical average of the parietal-occipital electrodes PO7 and PO8, according to the 10–10-system, was calculated and then subtracted from each individual EEG recording electrode (Jurcak et al., 2007).

We chose to analyze the above six reference sites and referencing techniques as three of them, A1/A2, average, and REST are commonly used in EEG practice (Yao, 2001), while the other three reference sites and referencing techniques, frontal (AFz), parietal (Pz) and parietal-occipital (PO7/PO8), although they are not commonly used in EEG practice, we thought to evaluate their suitability for CHEPS as, to our knowledge, they have not been evaluated in the literature.

### Subjects

For this study, we included 21 healthy subjects with a minimum age of 18 years: 10 males and 11 females. The data used in this manuscript is a subset taken out of a larger study and was collected between June 2018 and September 2019. The larger study was designed to investigate the level of small fiber neuropathy of patients with rheumatoid arthritis compared to a healthy control group. The EEG data subset used in this manuscript was extracted to answer a question of a purely technical nature (the ideal referencing technique to use when analyzing and displaying CHEPS), whereas the original study tests for a clinical hypothesis regarding differences in the somatosensory profile of patients with rheumatoid arthritis compared to a healthy control group. The clinical hypothesis of the original study does not collide or overlap with the technical hypothesis presented in this manuscript in any form. Additional data from the original study (e.g., the results of conditioned pain modulation or the results of quantitative sensory testing) and the data of the patients with rheumatoid arthritis is not presented in this publication as it is not needed to corroborate the hypothesis in this manuscript. At the time of publication of this manuscript, the original study had not been published.

We obtained written consent from all the participants. The local ethics committee approved the study procedures in a written statement (Ethics Committee Department of Medicine Goethe University Frankfurt, reference number 245/17). Furthermore, we conformed to the standards set by the Declaration of Helsinki. We asked the subjects to avoid taking any pain medication for five days before the commencement of the study visit. Exclusion criteria were the use of antidepressant medication, a history of alcohol abuse, and the presence of chronic pain or neuropathic diseases.

### Painful Stimuli

**Figure 1:**
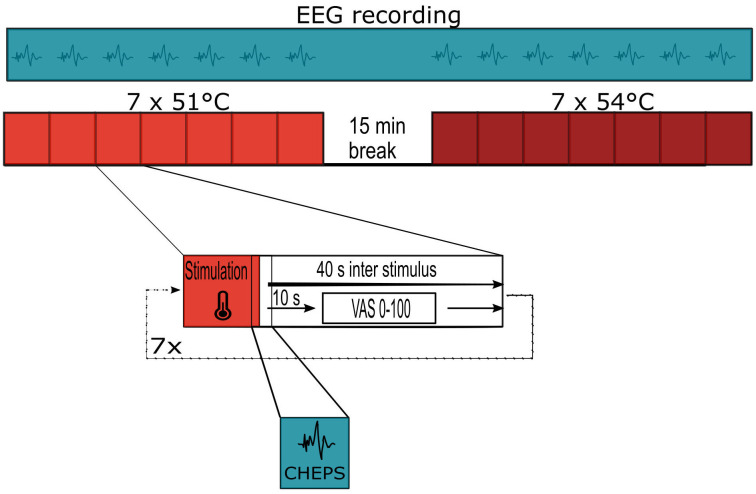
Study flow and stimulation pattern for each subject.

We outlined the study flow and stimulation pattern in Figure 1. We used the technique of CHEPS to apply painful stimuli to the left forearm of the subjects; seven stimuli were applied at 51°C and seven stimuli at 54°C using a MEDOC PATHWAY Pain and Sensory Evaluation System (Medoc Limited, Ramat Yishai, Israel). The temperature baseline of the thermal stimulation device was 32°C; this increased to the stimulation temperature of 51°C or 54°C at a rate of 70°C/s. We set the inter-stimulus interval to 40 s to minimize habituation (Bromm and Scharein, 1982). The thermode of the PATHWAY system was circular, with a diameter of 27 mm.

### EEG Recordings

Each study took place with the subject sitting, in a quiet room. The investigators equipped each subject with an EEG cap (g.Tec g.GAMMAcap^2^; Guger Technologies, Schiedlberg, Austria), which incorporated 21 active EEG electrodes (g.Tec g.SCARABEO) attached to a multichannel amplifier (g.Tec g.HIamp). The electrodes were placed in the standard configuration, following the 10-10 system; these were evenly distributed over the surface of the head. The ground electrode was attached to the subject’s forearm. We placed the Cz electrode midway between the nasion (the most anterior point on the nose) and inion (the squamous part of the occipital bone) and midway between both tragi (the small pointed eminence, visible on the external part of the ear). The use of active EEG electrodes guaranteed an exceptionally low output impedance, below 1 Ω, so that artifacts from the movement of the electrode cables were minimized (Metting van Rijn et al., 1990). We recorded the raw EEG using the g.Recorder software from g.TEC with a sample rate of 512 Hz. For visualization purposes only, we applied a high-pass filter at 1 Hz and a low-pass filter at 30 Hz. We set AFz as the initial reference site and stored the raw EEG recordings electronically.

## Methods

### EEG Analysis

We used EEGLAB, a MATLAB-based toolbox (The MathWorks Inc., Natick, MA, USA) for data preprocessing (Delorme and Makeig, 2004). We down-sampled the EEG to 256 Hz for data reduction using the EEGLAB’s *pop_resample* function. This function utilizes the resample-function from the MATLAB signal processing toolbox that automatically applies the necessary low-pass filter. Moreover, we high-pass filtered the data at 1 Hz (cutoff frequency: 0.5 Hz) and additionally low-pass filtered with a passband-edge at 40 Hz (cutoff frequency: 45 Hz) to eliminate 50 Hz line noise. For both bandpass filters, we used the EEGLAB *pop_eegfiltnew* function that applies a zero-phase bandpass filter to avoid phase shift. For preprocessing, we referenced the datasets to mathematically linked earlobes (A1/A2) by using the mathematical average of electrodes A1 and A2 as the reference signal for all other electrodes.

After adding the electrode locations to the datasets, we used Artifact Subspace Reconstruction (ASR) to detect and remove malfunctioning channels and to clean noisy data (Chang et al., 2018). We removed EEG channels if they did not correlate by at least 80% with their neighboring channels, for example, as a result of electrode displacement. In our case, our parameter selection led to a rejection of an average of 0.2 channels per dataset; the channel of interest for data inspection (Cz) was not rejected. We then interpolated all removed channels using spherical spline interpolation for the sole purpose of avoiding bias in the datasets that were later re-referenced to average or subjected to REST. For artifact rejection, we set the tolerance parameter for ASR to 20 (Chang et al., 2018). Thus, the variance of large-amplitude artifactual components (defined by PCA via the algorithm) is allowed up to a value of 20, compared to “clean” calibration data (i.e., the cleanest part of the recorded EEG data as defined by the algorithm; Mullen et al., 2015). This setting appeared to present a reasonable point of balance between rejecting data frames and correcting artifacts and eye-related components. The EEG dataset was then split into epochs using the 5V trigger from our thermal sensory testing device with a time range from −1 to +2 s around every event containing painful stimuli. This epoch range covers the whole duration of the stimulation; this takes approximately 629 ms from the baseline temperature (32°C) to the peak temperature (54°C) and back to the baseline temperature (32°C), with the peak temperature (54°C) being reached approximately 315 ms after stimulation onset.

For comparison, we created six different groups of datasets that solely differed in the definition of the reference. We kept one group of datasets at the A1/A2 reference while re-referencing the other datasets to AFz, Pz, average reference, REST (Dong et al., 2017), or mathematically linked PO7/PO8 (i.e., the mathematical average of electrodes PO7 and PO8). We then visualized and post-processed the epochs using MATLAB’s built-in functions. For calculation of the power frequency values, we used the EEGLAB *spectopo*-function that utilizes the *pwelch*-function of the MATLAB signal processing toolbox.

### Independent Component Analysis

We applied Independent Component Analysis (ICA) on the datasets with the reference set to AFz, A1/A2, average, and REST, using the integrated EEGLAB-function *runica* (Makeig et al., 2004a, b; Delorme et al., 2012; Pion-Tonachini et al., 2019). The rank for ICA decomposition was decreased by 1, accordingly, when we rejected an electrode or changed the reference of the dataset to REST or average.

Clustering approaches using EEGLAB’s *kmeans()* algorithm yielded in highly variable results. Hence, we decided to manually select ICs according to their event-related activity patterns to obtain functionally consistent clusters. We determined if a component was caused only by the painful contact heat stimulation according to its ERP waveform across trials with the help of the EEGLAB plugin *ICLabel* (Pion-Tonachini et al., 2019). Selection criteria were a visible deactivation and/or activation of the component with suitable latency around 500 ms, a visible peak in the delta region (<6 Hz) in the frequency diagram, and a visible *N*- and/or *P*-wave in the ERP spectrum. Scalp maps of the components were ignored during selection to avoid statistical double-dipping (Kriegeskorte et al., 2009). The independent components were evaluated in a blinded fashion: the IC images were provided with a four-digit code by author BA and then evaluated by author MA according to the mentioned criteria. MA was blinded to the subject code, IC scalp maps, and the IC number. We then clustered the manually selected components using EEGLAB’s *STUDY* function. If we selected more than one component per subject, we assigned the component with the highest amplitude to the cluster and discarded all others for further evaluation.

### Statistical Analyses

We applied different statistical approaches to fully describe our data. To present visually the EEG reaction to the stimuli, we used a five-point moving mean to smooth the response. Because of our rather small sample size, we decided to apply non-parametric tests for inferential statistics. For an overview of group differences between the different referencing approaches, we applied the Friedman test using the MATLAB *friedman* function. For the inference statistics, the significance level was set to *p* < 0.05. For *Post hoc* analysis, we calculated the Wilcoxon signed-rank test with a Bonferroni correction. For comparison of the average scalp maps, we used the Wilcoxon rank-sum test with significance thresholds of 0.05, 0.01, and 0.001. We have only reported results as being significant if we observed a cluster of significant results, similar to the idea of cluster-based permutation tests (Sassenhagen and Draschkow, 2019).

## Results

### Overview

One female subject expressed discomfort while wearing the EEG cap throughout the study; as the study protocol was not finished, we excluded this subject’s data for analysis. All remaining 20 subjects (10 male, mean age 56.30 ± 14.66 years; 10 female, mean age 54.90 ± 14.40 years) showed visible *N*- and *P*-waves at both stimulation settings (51°C and 54°C stimulation temperatures) in one or more stimulation epochs. We visually compared the average CHEPS signal obtained with the 51°C and 54°C stimulation temperatures. We observed comparable reactions in terms of *N-*wave delay, *P*-wave delay, *N*-*P*-wave duration, and overall ERP waveform, but lower magnitudes for the *N*-*P*-wave at 51°C when compared to the 54°C stimulation temperature. Therefore, we have presented the findings from the 54°C experiments in the main text. For completeness, we have presented the average ERP waveforms at the 51°C stimulation temperature in Supplementary Figure 1.

### Influence of the Referencing Technique on the EEG Stimulus-Response

**Figure 2:**
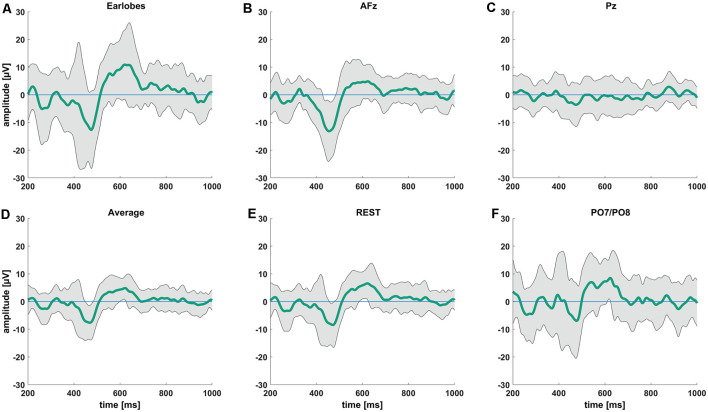
Average contact heat evoked potentials (CHEPS) waveforms from 200 ms to 1,000 ms following the stimulus at 54°C stimulation temperature. The green line indicates the average amplitude at the Cz electrode location with **(A)** mathematically linked earlobes A1/A2, **(B)** AFz, **(C)** Pz, **(D)** average, **(E)** REST, and **(F)** mathematically linked PO7/PO8 as the reference sites.

We observed the highest average *P*-peak amplitude with the A1/A2 electrodes set as the reference site, while the lowest average *N*-peak amplitude was observed with the AFz electrode set as the reference site. Both the average reference and REST showed smaller average *N*- and *P*-wave amplitudes with smaller standard deviation windows (gray areas in Figure 2) compared to the A1/A2 reference. The PO7/PO8 reference electrodes showed rather small visible *N*- and *P*-peaks with an overall distorted waveform, increased standard deviation, and visible alpha waveforms (i.e., a visible EEG signal with a frequency between 8–12 Hz) compared to the EEG signals with other reference electrode sites or techniques. The use of the Pz reference led to no identifiable *N*- and *P*-peaks. Figure 2 shows the CHEPS waveforms at our designated Cz electrode site with the applied moving mean for the different EEG reference settings. Supplementary Table 1 contains detailed amplitude information.

After visually inspecting Figure 2, we discarded Pz and PO7/PO8 as possible reference sites for the evaluation of CHEPS and so these were not considered for further analyses. With regards to Pz as the reference site, we could not properly identify N and P peaks because the EEG signal appeared too close to the baseline. Concerning PO7/PO8 as the reference site, although we calculated *N*- and *P*-values for every subject in Table 1 and the *N*- and *P*-wave delays in Table 2, we did not evaluate the reference site further, as the CHEPS waveform in Figure 2 appeared distorted, overall.

**Table 1:** *N*-, *P*-, and *N*-*P*-wave amplitudes for every reference site for the 54°C stimulation temperature and their respective standard deviations.

	*N-*wave amplitude [μV]	*P*-wave amplitude [μV]	*N*-*P*-wave [μV]	Percentage of amplitude compared to A1/A2 (assigning 100% to A1/A2) [%]
A1/A2	−25.13 ± 15.08	21.83 ± 7.81	46.96 ± 19.25	100
AFz	−17.58 ± 9.42	13.12 ± 5.70	30.71 ± 12.38	68.87 ± 29.23
Pz	No value	No value	No value	No value
Average	−13.11 ± 6.33	10.09 ± 3.80	23.20 ± 8.40	49.96 ± 9.76
Rest	−15.50 ± 8.52	12.78 ± 4.50	28.28 ± 10.98	60.32 ± 7.08
PO7/PO8	−19.46 ± 10.59	19.38 ± 8.32	38.85 ± 14.41	88.10 ± 25.69

*The data have been calculated for every subject individually, using a window from 250 ms to 550 ms following the stimulus to detect the lowest peak (*N-*wave) and a window from 550 ms to 800 ms to detect the highest peak (*P*-wave). The amplitude of the *N*-*P*-wave, as a percentage of amplitude at the earlobes (assigned 100%), is also presented. The *N*-*P*-wave value for every subject is presented in the supplement*.

**Table 2:** *N*- and *P*-wave delays and *N*-*P*-wave duration for every reference site for the 54°C stimulation temperature and their respective standard deviations.

	*N-*wave delay [ms]	*P*-wave delay [ms]	*N*-*P*-wave duration [ms]
Earlobes	431.25 ± 74.19	641.80 ± 77.52	210.55 ± 98.85
AFz	432.42 ± 73.92	661.52 ± 92.81	229.10 ± 114.43
Pz	No value	No value	No value
Average	418.75 ± 80.87	666.99 ± 83.07	248.24 ± 105.57
Rest	433.20 ± 66.12	641.60 ± 77.92	208.40 ± 94.89
PO7/PO8	376.76 ± 87.75	622.46 ± 76.46	245.70 ± 106.08

*The data have been calculated for every subject individually, using a window from 250 ms to 550 ms following the stimulus to detect the lowest peak (N-wave) and a window from 550 ms to 800 ms to detect the highest peak (*P*-wave)*.

**Figure 3:**
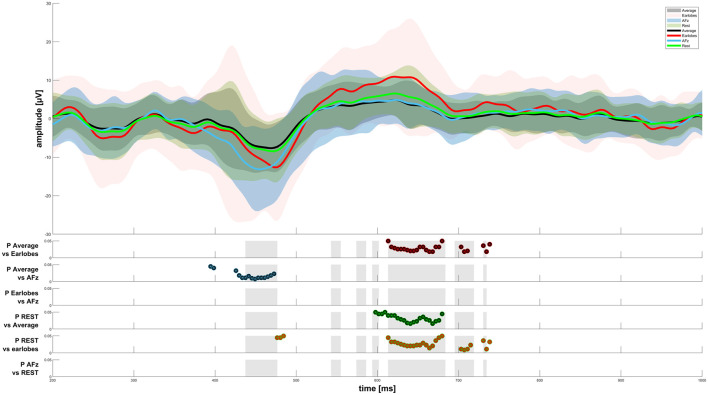
CHEPS graphs for four reference sites. The results of the Friedman test were plotted as gray areas in the second half of the figure. *Post hoc* tests with a significance level *p* < 0.05 have been plotted for every reference site comparison.

When analyzing the amplitudes in the EEG response, we found significant differences between the average reference, A1/A2, REST, and AFz reference settings, as displayed in Figure 3. The AFz setting led to significantly higher amplitudes in the *N*-peak when compared to the average reference. We also observed a significantly higher *P*-peak with A1/A2 as the references when compared to the average or REST referencing technique. Interestingly, the *P*-peak of the CHEPS waveform with the REST referencing technique was significantly higher than the *P*-peak with the average reference, although the difference in absolute terms is scarcely noticeable. Thus, although our testing revealed a “statistically significant difference”, this should not be taken as advice that there is indeed a relevant difference in CHEPS waveform between those two referencing techniques. In practical terms, both average and REST may perform equally in terms of the observed response (Amrhein et al., 2019).

**Figure 4:**
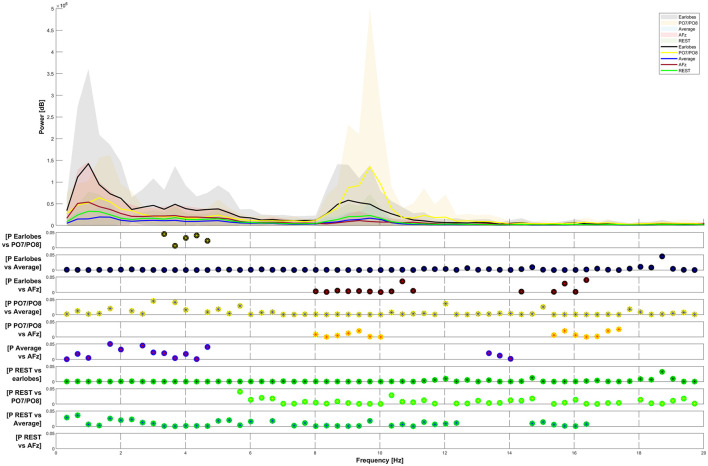
Frequency-power plots for five selected reference sites. The Friedman test shows significance (*p* < 0.05) for all observed differences. *Post hoc* analysis (Wilcoxon signed-rank with Bonferroni correction) has been plotted for data pairs in the lower half of the figure.

The spectral power of the response also heavily depended on the choice of the reference point. We have presented the spectral power for the five chosen reference sites from 0–20 Hz in Figure 4. We do not show spectral graphs for the Pz reference site due to the reasons outlined above. For comparison, we present in Figure 4 the spectral graph for PO7/PO8 set as a reference site to emphasize the alpha noise (visible parts of the EEG signal with a frequency of 8–12 Hz) that occurs when choosing occipital reference sites. The CHEPS signal with PO7/PO8 set as the reference site shows the highest average peak in the alpha region (8–12 Hz) that statistically differs from all other reference sites, except for the earlobes reference point (*p* < 0.05). This statistical difference in the power spectrum is also observable in the amplitude-time spectrum via visual inspection.

In Figure 4, the Friedman test indicated a significant difference in the comparison of the five plotted graphs for all frequencies between 0 and 20 Hz. The *Post hoc* testing revealed that the power of the distribution between the A1/A2 reference and the PO7/PO8 reference, as well as between the REST and the AFz reference, was not significantly different in most frequency ranges (*p* > 0.05).

### Scalp Maps of Independent Components

**Figure 5:**
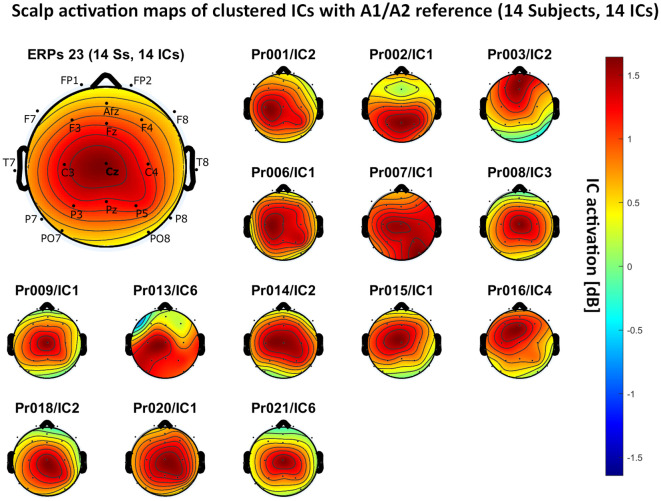
Scalp activation for the selected independent components (ICs) for 14 subjects with A1/A2 as the reference site. The color-coding indicates the activation of the components in dB. The average activation is plotted in the top-left corner, including detailed electrode locations.

**Figure 6:**
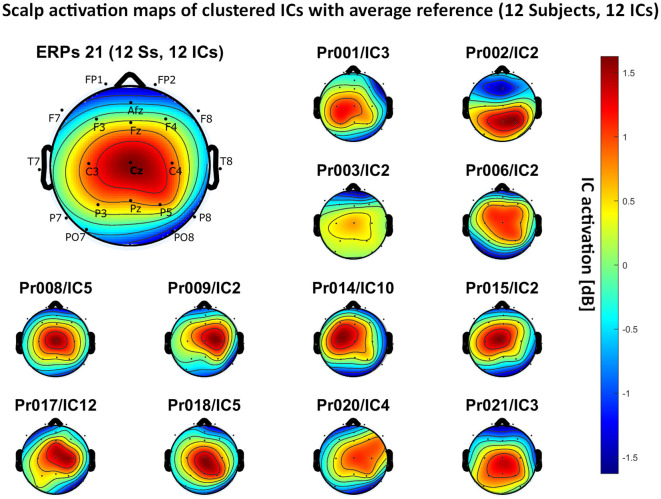
Scalp activation for the selected ICs for 12 subjects with average reference as referencing technique. The color-coding indicates the activation of the components in dB. The average activation is plotted in the top-left corner, including detailed electrode locations.

**Figure 7:**
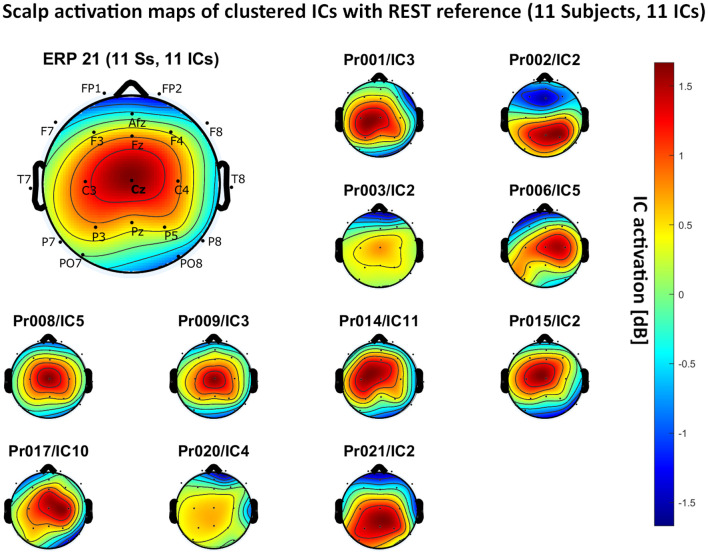
Scalp activation for the selected ICs for 11 subjects with REST as a referencing technique. The color-coding indicates the activation of the components in dB. The average activation is plotted in the top-left corner, including detailed electrode locations.

**Figure 8:**
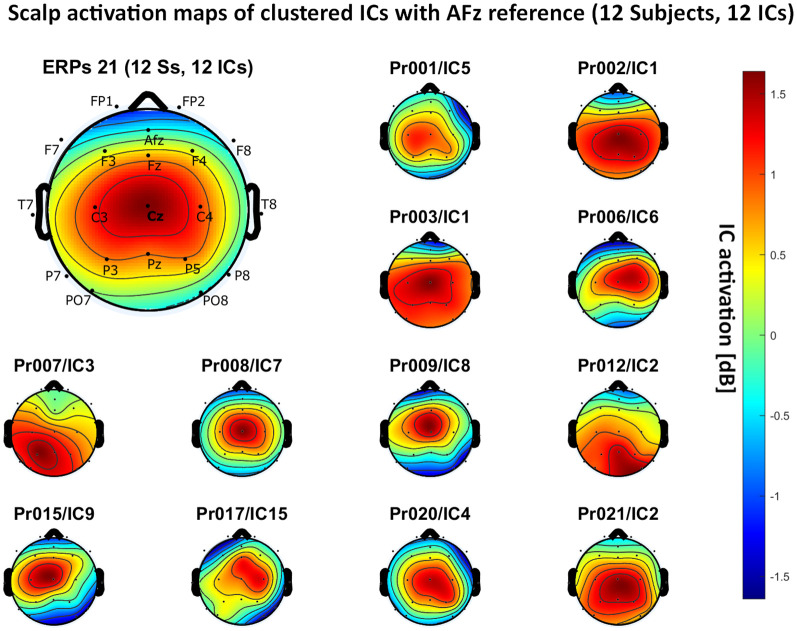
Scalp activation for the selected ICs for 12 subjects with AFz as the reference site. The color-coding indicates the activation of the components in dB. The average activation is plotted in the top-left corner, including detailed electrode locations.

For the different reference sites, we found the following numbers of independent components (ICs) that could be directly allocated to our painful stimulation:

– A1/A2 reference: 14 independent components, see Figure 5.
– average reference: 12 independent components, see Figure 6.
– REST reference: 11 independent components, see Figure 7.
– AFz reference: 12 independent components, see Figure 8.

**Figure 9:**
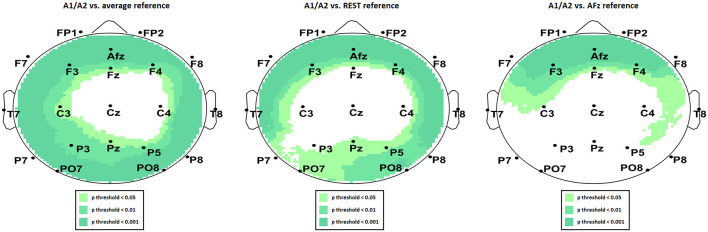
Statistical comparison of average component activation between A1/A2 as the reference site and average (left), REST (middle), and AFz (right) as referencing technique/site.

We present the scalp map of every selected component for each reference site and their average scalp map in Figures 5–8. We statistically compared the average scalp maps in Figure 9 in a binary fashion, using the Wilcoxon rank-sum test with significance thresholds of 0.05, 0.01, and 0.001; we did not observe any clusters of statistical differences at any thresholds between the REST, average, and AFz reference sites. Consequently, we have not shown the statistical comparison of these reference sites. Concerning the A1/A2 reference, we observed that this reference site exhibited significant differences at all thresholds compared to the other three reference sites. While the activation of the noxious contact heat-related component between A1/A2 and AFz as the reference sites only exhibited significant differences in the frontal head region, significant differences between A1/A2 and REST or average, respectively, are particularly evident in a circular fashion around the whole head.

As far as the average and REST referencing techniques are concerned, the extent of noxious CHEPS (i.e., the magnitude of the *N*- and *P*-waves) depends on the number of electrodes used for measurements and where they are placed. According to Figures 6, 7, the CHEPS amplitude would rise if more electrodes were placed on the outer regions of the head (as those electrodes do not pick up the specific CHEPS activation). Subsequently, if more electrodes were placed next to Cz, a region, where still a reasonable level of CHEPS IC activation is picked up, then setting the reference to the average or REST referencing technique would result in lower amplitudes of the CHEPS waveform. Hence, when measuring noxious CHEPS following our experimental setup and selecting the REST or average as referencing technique, the exact positioning and the number of electrodes used are key factors for the strength of the resulting response (mainly the *N*-wave and *P*-wave magnitudes).

**Figure 10:**
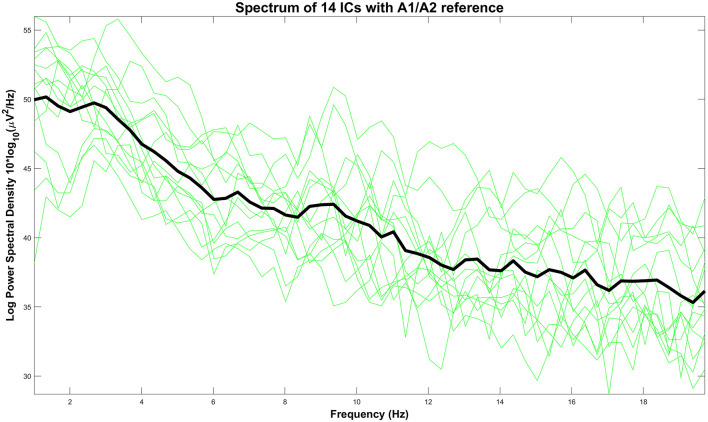
Power spectrum of every IC with A1/A2 as the reference site (green lines) and their average (black line).

We have presented the spectral power of the pain-related clustered ICs with the A1/A2 referencing point in Figure 10. On average, we observed the highest absolute power in the lower frequency regions (<6 Hz) with peaks around 1 Hz and 3 Hz. Based on this fact, our settings for high-pass filtering (which was set to 1 Hz) probably also influence the CHEPS waveform and amplitude. Our results are, thus, only applicable to our chosen filter settings (1 Hz passband-edge). We wish to highlight that other common physiological EEG characteristics, such as alpha and beta waves above 6 Hz, can be seen as physiological artifacts that tend to distort waveforms if those signals are recorded either at the reference site or the measurement site.

## Discussion

We hypothesized that single (AFz or Pz) or dual (PO7/PO8 or A1/A2) reference electrode locations would have practical advantages over referencing techniques that require more electrodes that are evenly distributed over the head surface. Examples for the latter are both average and REST, with REST also requiring the computation of a lead field matrix. High-density layouts are, however, not necessary requirements for measuring CHEPS, so we evaluated if one of the single or dual-electrode locations excels when analyzing CHEPS in the amplitude-time spectrum of the EEG.

As described in the results, we observed poor responses for the PO7/PO8 reference site and the Pz reference setting. Regarding Pz as the reference site, Figures 5–8 show that the Pz electrode always records a high amount of the pain-related independent component, regardless of which reference was chosen. This has led to the conclusion that Pz also appears to record a certain amount of the CHEPS amplitude so that when referencing Cz vs. Pz, the overall CHEPS amplitude is mathematically canceled out. As a conclusion, Pz should not be chosen as a reference site in studies that aim to record painful CHEPS.

In Figure 4, we outlined that the reference site PO7/PO8 leads to the greatest amount of alpha power in the data. This alpha power is visible, even in the average EEG amplitude-time spectrum in Figure 2 (graph F), and distorts the CHEPS waveform in a manner that renders identification of the *N-*wave and *P*-wave demanding. The alpha power increases due to activity in the primary visual cortex when subjects close their eyes (Britton et al., 2016). In conclusion, PO7/PO8 should not be used as a reference site to avoid having increased amounts of physiological alpha noise present in the data.

AFz as a frontal reference site commonly picks up a high amount of eye blink artifacts that share the same frequency characteristics as the CHEPS waveforms in the regions below 6 Hz (Dimigen, 2020). In our study, AFz worked reasonably well as a reference site as we used ASR to clean up the ocular artifacts to a certain extent. However, no artifact rejection mechanism is perfect. In the average CHEPS waveform with AFz as the reference, we were not able to determine how much noise by eye blinks was still present in the data, even after artifact rejection. Therefore, we would not recommend AFz as a frontal reference site for the CHEPS experiments.

Following visual analysis of Figures 2, 3, we concluded that the average and REST referencing techniques both worked reasonably well to display noxious CHEPS in the averaged EEG amplitude-time spectrum. These referencing techniques require an electrode layout that covers the whole head and is evenly distributed, however, the extensive layout of electrodes is not a mandatory requirement when investigating CHEPS. On the other hand, for further analysis, such as Dipole Source Localization, the REST or average referencing techniques might be necessary (Trujillo et al., 2017). There have been attempts to converge protocols to reference-free techniques in recent literature, although it has been highlighted that no reference can be ideal for all EEG recordings (Kayser and Tenke, 2010). As far as our test sample showed, modern techniques such as REST are not necessary for a simple research of noxious CHEPS in the amplitude-time spectrum, as other options may perform better. Practical reasons (such as the number of electrodes required) and the ease of the intercomparison of results between studies should be considered when designing an experiment and selecting a referencing technique; nonetheless, the theoretical advantages of some techniques have been highlighted in recent studies (Yao et al., 2019). Overall, although the use of the average and REST referencing techniques result in visible CHEPS amplitudes, their pitfalls, in terms of practicability and ICA performance, at least in our case, mean that they cannot be generally recommended if the aim of the study is simply to analyze the amplitude-time spectrum of the EEG.

Figures 5–8 also point out that Cz is the electrode location where, on average, the signal of the noxious contact heat-related ICs is the highest, regardless of which reference site is chosen. A visual interpretation of the average scalp heat map in those figures reveals that the average IC activation power at the Cz electrode location has the highest magnitude (around 1.5 dB), compared to the IC activation power at every other electrode location. For example, the power at electrode location Fz, Pz, C3, and C4, which in our electrode layout were the locations closest to Cz, tended to fluctuate around 1 dB. As higher IC ERP activation power correlates with a higher visible CHEPS amplitude, Cz should be chosen as the measurement site.

Concerning the ICA performance, A1/A2 as a reference site enabled us to identify pain-related ICs in 14 out of the 20 subjects, with other reference settings resulting in inferior performances. By its nature, ICA has a bias towards high amplitude data and cannot recover the exact amplitude of the dipole generator which is in our case responsible for eliciting the CHEPS waveform in the amplitude-time spectrum of the EEG (Debener et al., 2010). As the A1/A2 reference site resulted in the highest overall CHEPS amplitudes, the ICA performed best in our test data. However, ICA should not be used to compare the CHEPS amplitude, as no component would fully include the whole amplitude that is generated by the dipole that outputs the pain-related EEG information in the head. In our example, ICA only served as a technique to identify the frequency regions that the CHEPS amplitudes appeared in the spectrogram and at which electrode sites the signal can be visualized.

We also wish to highlight that the three recent studies that published normative data for CHEPS all used A1/A2 as the reference (Granovsky et al., 2016; Jutzeler et al., 2016; Rosner et al., 2018). Study (Granovsky et al., 2016) evaluated the same body region with the same stimulation pattern as our study. Study (Jutzeler et al., 2016) and (Rosner et al., 2018) evaluated different body regions with a slightly different stimulation pattern and a different baseline temperature. All three studies analyzed the same CHEPS parameters as we did in this manuscript, such as *N-*wave delay, *P*-wave delay, and *N*-*P*-wave. Hence, the results of CHEPS studies (i.e., between different body regions or different baseline temperatures) can only be compared with published normative data if the reference site in future CHEPS studies is the same (i.e., A1/A2). We strongly recommend that future studies additionally record data from both earlobes during data collection; this would allow for the data comparison of their data with published normative data, even if the reference is subsequently changed for further analysis. This would pave the way towards a more standardized use of the EEG in the research of noxious CHEPS.

In conclusion, there is no optimum reference point for all EEG studies. The results of this research are, thus, only applicable to common pain-related brain dynamics (CHEPS). By using A1/A2 as the reference site, we found the *N*- and *P*-wave amplitudes in every subject to be higher than in all the other referencing settings. Also, the limited requirements of using A1/A2, in terms of practical implementation, meant that it was easier to identify and evaluate the *N*- and *P*-waves and, thus, improve the performance of the ICA. Future studies should agree on A1/A2 as the reference site, as methodological standardized recordings will foster the role of CHEPS in pain research. The technique can then be incorporated into clinical research that tests for differences in pain profiles between groups, i.e., patients with small fiber neuropathy vs. healthy control subjects.

## Data Availability Statement

The raw data supporting the conclusions of this article will be made available by the authors, without undue reservation.

## Ethics Statement

The studies involving human participants were reviewed and approved by Ethics Committee Department of Medicine Goethe University Frankfurt Theodor-Stern-Kai 7 Haus 1, 2. OG, Zimmer 207-211 60590 Frankfurt am Main Reference number: 245/17. The patients/participants provided their written informed consent to participate in this study.

## Author Contributions

MA was responsible for the design of the study, data collection, data analysis, creation of data plots, statistical analysis, and writing of the first draft. BA was responsible for the design of the study, subject recruitment, and data collection. MK was responsible for data analysis, creation of data plots, and statistical analysis. SZ was responsible for image processing, data analysis, and creation of data plots. CW was responsible for the design of the study, data management, and funding. All authors contributed to the article and approved the submitted version.

## Conflict of Interest

The authors declare that the research was conducted in the absence of any commercial or financial relationships that could be construed as a potential conflict of interest.
