# Supplementary material for: Application of Referencing Techniques in EEG-Based Recordings of Contact Heat Evoked Potentials (CHEPS)
Source: Front Hum Neurosci. 2020 Dec 2;14:559969. doi: 10.3389/fnhum.2020.559969 (PMC7738344; doi:10.3389/fnhum.2020.559969)
Supplement: Supplementary file 1 [file Data_Sheet_1.docx]

Supplementary Material

# Supplementary Figures and Tables

## Supplementary Figures

**
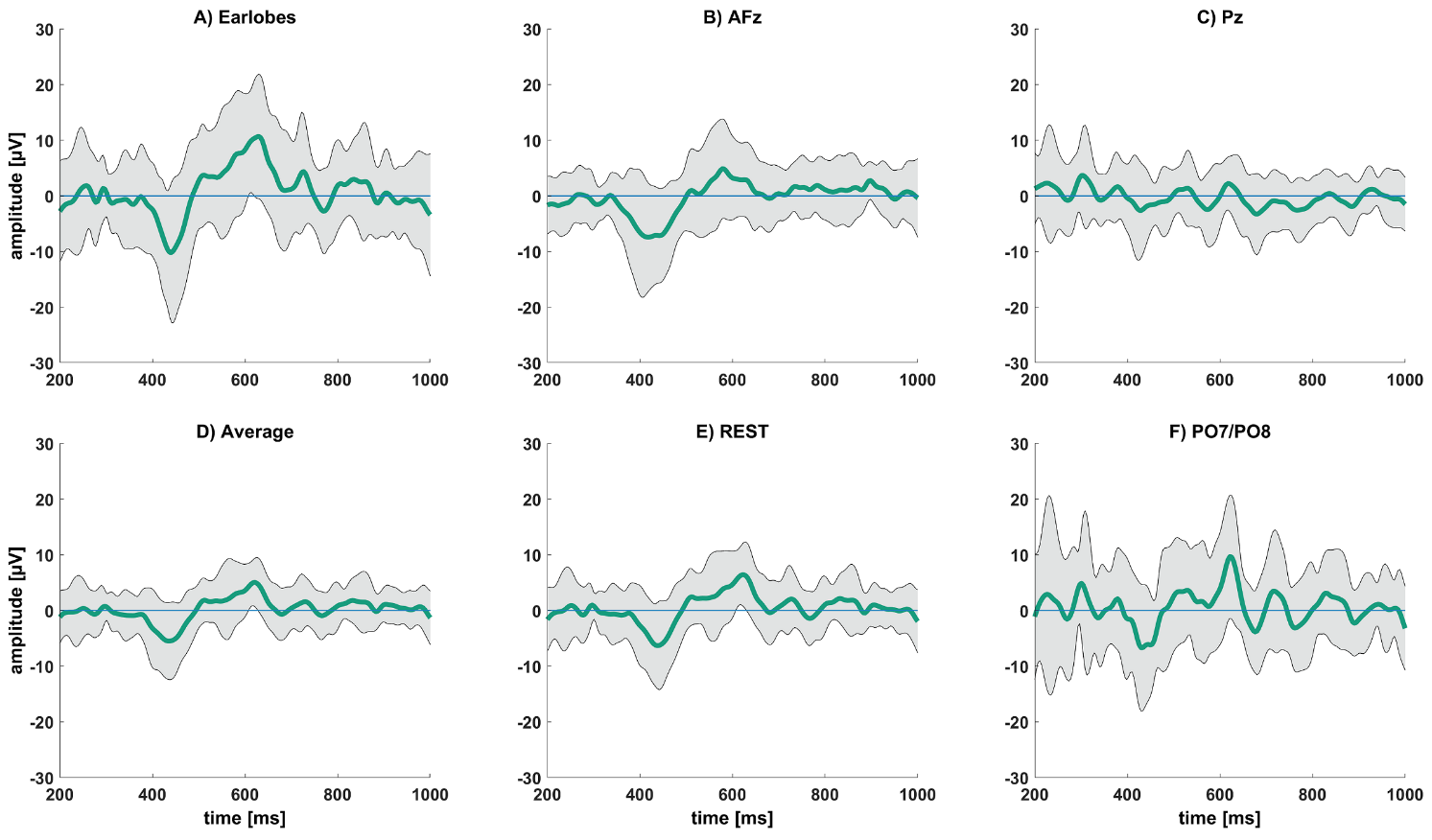
**

**Supplementary Figure S1.** Average PREP amplitudes at Cz electrode location for 51 °C stimulation temperature

## Supplementary tables

| Subject No. | A1/A2 [µV] | AFz  [µV] | PO7­PO8 [µV] | Average [µV] | REST [µV] |
| --- | --- | --- | --- | --- | --- |
| 1 | 35.87 | 26.96 | 22.02 | 24.31 | 29.13 |
| 2 | 51.81 | 53.44 | 24.89 | 29.91 | 22.16 |
| 3 | 42.39 | 29.68 | 22.30 | 25.15 | 43.19 |
| 4 | 35.05 | 25.52 | 21.49 | 24.56 | 50.41 |
| 5 | 47.39 | 41.52 | 26.23 | 30.04 | 42.51 |
| 6 | 62.03 | 44.58 | 32.22 | 36.17 | 42.49 |
| 7 | 53.57 | 34.78 | 33.14 | 36.53 | 44.78 |
| 8 | 34.84 | 20.27 | 16.02 | 20.85 | 25.78 |
| 9 | 34.56 | 32.92 | 21.15 | 24.36 | 42.83 |
| 10 | 29.28 | 28.73 | 17.06 | 19.26 | 26.57 |
| 11 | 29.25 | 46.08 | 18.74 | 17.91 | 34.60 |
| 12 | 31.70 | 10.99 | 13.28 | 18.22 | 33.39 |
| 13 | 38.09 | 21.89 | 15.75 | 21.28 | 31.56 |
| 14 | 53.76 | 31.21 | 29.40 | 35.31 | 41.95 |
| 15 | 59.97 | 17.61 | 24.67 | 29.73 | 50.01 |
| 16 | 72.26 | 34.90 | 30.02 | 48.08 | 71.99 |
| 17 | 10.97 | 4.80 | 3.06 | 4.73 | 12.99 |
| 18 | 62.70 | 28.83 | 25.74 | 34.78 | 29.92 |
| 19 | 54.03 | 30.16 | 24.20 | 30.50 | 31.99 |
| 20 | 99.65 | 49.23 | 42.62 | 53.94 | 68.69 |
|  |  |  |  |  |  |
| Mean | 46.96 | 30.71 | 23.20 | 28.28 | 38.85 |
| SD | 19.25 | 12.38 | 8.40 | 10.98 | 14.41 |

**Supplementary Table S1.** Values for the N-P-wave at Cz electrode location (54°C stimulation temperature) for every subject at every reference site, excluding Pz. Values in [µV].
